# Prediction of long-term outcomes of HIV-infected patients developing non-AIDS events using a multistate approach

**DOI:** 10.1371/journal.pone.0184329

**Published:** 2017-09-08

**Authors:** Mar Masiá, Sergio Padilla, Santiago Moreno, Xavier Barber, Jose A. Iribarren, Jorge del Romero, Juan L. Gómez-Sirvent, María Rivero, Francesc Vidal, Antonio A. Campins, Félix Gutiérrez, Cohorte de la Red de Investigación en Sida (CoRIS)

**Affiliations:** 1 Infectious Diseases Unit, Hospital General de Elche, Universidad Miguel Hernández, Alicante, Spain; 2 Infectious Diseases Service, Hospital Ramón y Cajal, IRYCIS, Madrid, Spain; 3 Statistics, Centro de Investigación Operativa, Universidad Miguel Hernández, Elche, Alicante, Spain; 4 Hospital Universitario Donostia, San Sebastián, Spain; 5 Centro Sanitario Sandoval, Madrid, Spain; 6 Hospital Universitario de Canarias, Santa Cruz de Tenerife, Spain; 7 Hospital de Navarra, Pamplona, Spain; 8 Hospital Universitario Joan XIII, Tarragona, Spain; 9 Hospital Universitario Son Espases, Palma de Mallorca, Spain; 10 Instituto de Salud Carlos III, Madrid, Spain; Fundacao Oswaldo Cruz, BRAZIL

## Abstract

**Objetives:**

Outcomes of people living with HIV (PLWH) developing non-AIDS events (NAEs) remain poorly defined. We aimed to classify NAEs according to severity, and to describe clinical outcomes and prognostic factors after NAE occurrence using data from CoRIS, a large Spanish HIV cohort from 2004 to 2013.

**Design:**

Prospective multicenter cohort study.

**Methods:**

Using a multistate approach we estimated 3 transition probabilities: from alive and NAE-free to alive and NAE-experienced (“NAE development”); from alive and NAE-experienced to death (“Death after NAE”); and from alive and NAE-free to death (“Death without NAE”). We analyzed the effect of different covariates, including demographic, immunologic and virologic data, on death or NAE development, based on estimates of hazard ratios (HR). We focused on the transition “Death after NAE”.

**Results:**

8,789 PLWH were followed-up until death, cohort censoring or loss to follow-up. 792 first incident NAEs occurred in 9.01% PLWH (incidence rate 28.76; 95% confidence interval [CI], 26.80–30.84, per 1000 patient-years). 112 (14.14%) NAE-experienced PLWH and 240 (2.73%) NAE-free PLWH died. Adjusted HR for the transition “Death after NAE” was 12.1 (95%CI, 4.90–29.89). There was a graded increase in the adjusted HRs for mortality according to NAE severity category: HR (95%CI), 4.02 (2.45–6.57) for intermediate-severity; and 9.85 (5.45–17.81) for serious NAEs compared to low-severity NAEs. Male sex (HR 2.04; 95% CI, 1.11–3.84), age>50 years (1.78, 1.08–2.94), hepatitis C-coinfection (2.52, 1.38–4.61), lower CD4 cell count at cohort entry (HR 2.49; 95%CI 1.20–5.14 for CD4 cell count below 200 and HR 2.16; 95%CI 1.01–4.66 for CD4 cell count between 200–350, both compared to CD4 cell count higher than 500) and concomitant CD4<200 cells/mL (2.22, 1.42–3.44) were associated with death after NAE. CD4 count and HIV-1 RNA at engagement, previous AIDS and hepatitis C-coinfection predicted mortality in NAE-free persons.

**Conclusion:**

NAEs, including low-severity events, increase prominently the risk for mortality in PLWH. Prognostic factors differ between NAE-experienced and NAE-free persons. These findings should be taken into account in the clinical management of PLWH developing NAEs and may permit more targeted prevention efforts.

## Introduction

Life expectancy has dramatically improved in people living with HIV (PLWH) since the introduction of combination antiretroviral therapy (ART) [1]. Opportunistic infections and HIV-related malignant diseases have been replaced by a variety of comorbid conditions, also known as non-AIDS events (NAEs), as major causes of morbidity and mortality in PLWH taking suppressive ART [2,3]. A high prevalence of comorbidity, ranging from 34% to 70% in persons older than 65 years has been described in the HIV population [4,5], as well as incidence rates as high as 29 NAEs per 1000 person-years [6].

The spectrum of comorbidities is similar to that seen in the general population, although some serious events like hepatic, malignant, renal, and cardiovascular-related diseases have been found to occur more frequently in PLWH than in their uninfected pairs [3, 7–9]. As a consequence, serious NAEs and the associated risk factors and outcomes have become in recent years an area of interest of researchers [3, 6, 10–12]. However, the most frequent NAEs recorded in the HIV population consist of non-severe events, like bacterial pneumonia, psychiatric diseases, bone fractures, or diabetes [4–6]. In contrast to severe events, there is limited information about outcomes associated with these less serious comorbidities commonly diagnosed in PLWH [13, 14].

Multi-state models are a useful tool to analyze survival when transient states are identified [15]. In PLWH, multistate models have been used to characterize HIV disease progression [16, 17], or to estimate the incidence of HIV infection by combining HIV and AIDS surveillance data [18]. However, the impact of the prognostic factors in progression from a state in which no NAE has been occurred (NAE-free) to a state where any NAE (NAE-experience) and death has occurred have not yet characterized by multistate models. We aimed to describe the clinical outcomes of PLWH after experiencing any of the most frequent NAEs, to classify NAEs according to their impact on mortality, and to assess the prognostic factors associated with mortality in PLWH who had developed any first incident NAE, and also in NAE-free PLWH, in a contemporary cohort using a multi-state approach.

## Methods

The cohort of adults with HIV infection of the AIDS Research Network (CoRIS) is an open, prospective, multicentre cohort of subjects with confirmed HIV infection, naïve to ART at study entry, who are recruited in HIV care units of the Spanish Public Health System [19]. CoRIS was launched in 2004. Each centre recruits into the cohort all subjects seen for the first time who meet the following criteria: over 13 years of age, confirmed HIV diagnosis, and naive to ART. Written informed consent is obtained from all PLWH. Demographic, clinical, laboratory, microbiological and treatment information is recorded in a central electronic database, based on a structured event reporting form containing the list of events to be reported and the precise definition of each NAE required for the inclusion (see S1 Annex), which is regularly updated [6]. For the present study, only the first event occurring after cohort engagement was included in the analysis. All PLWH were followed-up from the inclusion date (first visit at centre) until death, cohort censure (October 31^st^, 2013) or loss to follow-up, defined as no information provided during the last year, and no evidence of death. For ascertainment of classification, CoRIS database was cross-checked with the National Death Index.

Approval from each hospital’s Ethics Committee, including the Ethics and Clinical Research Committee of Hospital General de Elche, Hospital Ramón y Cajal, Hospital Universitario Donostia, Hospital Universitario de Canarias, Santa Cruz de Tenerife, Hospital de Navarra, Pamplona, Hospital Universitario Joan XIII, Tarragona, and Hospital Universitario Son Espases, Palma de Mallorca; and written informed consents from the patients were obtained.

### Non-AIDS events definition and classification

All centres were invited in February 2008 to provide the following incident NAEs: cardiovascular-related (acute myocardial infarction, angina, congestive heart failure, stroke, transient ischemic attack, silent cerebrovascular disease, peripheral arterial disease, coronary-related death), non-AIDS-defining malignancies, renal-related (acute renal failure, chronic kidney disease, renal tubulopathy/Fanconi syndrome, permanent dialysis, kidney biopsy), liver-related (ascites, hepatic encephalopathy, variceal haemorrhage, hepatic transplant, hepatocellular carcinoma, liver insufficiency/cirrhosis), psychiatric (depression requiring drug therapy, suicide attempt, psychosis diagnosed by a psychiatrist), bone-related (non-traumatic vertebral and non-vertebral fractures, avascular necrosis of the bone), non-*Pneumocystis jiroveci* pneumonia, and metabolic (diabetes, lactic acidosis). Centres were asked to collect retrospectively all of the above NAEs occurring from the day of entry in the cohort to February 2008, and to report them prospectively since then.

Death due to an AIDS-defining event was defined as death attributable to a category C disease listed by the CDCs [20]. Death due to a NAE was classified according to a revised version of the “Coding Death in HIV” (CoDe) classification system [21,22]. All-cause death event was defined as death due to any known or unknown cause. Non-AIDS events that directly resulted in death counted only as outcome and did not count as exposure variable. NAEs were initially categorized according to severity into three groups (low-severity, intermediate-severity and serious events) by using a modified Charlson Comorbidity Index [23].

### Statistical analyses

The baseline characteristics were summarized using descriptive statistics. For handling missing data, we applied the “available-case analysis” method, which uses all available data to compute each statistic. Longitudinal data from all PLWH from the inclusion date until death, cohort censoring or loss to follow-up were computed to estimate the incidence rates of NAE and death per 1000 person-years of follow-up. Incidence rate ratios (IRR) were calculated by median-unbiased estimation, and unconditional maximum likelihood estimation. Ninety five percent confidence intervals were calculated using exact methods, and normal approximation. Differences in the proportion of the causes of death between NAE-free and NAE-experienced PLWH were assessed using a Pearson’s chi-square test for independence.

The main data analysis was performed using an illness-death multi-state model [24, 25]. Multi-state models generalize competing risks analysis by taking into account the sequences of multiple events for each patient and can estimate the change in the risk of each event after the occurrence of other events. In other words, multi-state models could be used if we want to predict how the occurrence of an event changes the disease course. We built an “illness-death” form of multi-state model considering a transition matrix that evaluated the movement of PLWH in the following states: (1) the starting status, alive and NAE-free; (2) the transitional state, alive and NAE-experienced, and (3) a final state of all-cause death. Three transition paths were permitted: (1) from alive and NAE-free to alive with NAE, named “NAE development”; (2) from alive and NAE-free to death, named “Death without NAE”, and (3) from alive and NAE-experienced to death, named “Death after NAE”. Counting time process format was used to model time to the occurrence of NAEs or deaths. Likelihood ratio test was the test used for comparing the goodness of fit of models against the null model. All relevant data are within the paper and its Supporting Information files. The multi-state model analysis was carried out using the *mstate* R-package [26]. To check the proportionality of effects, we used the Schoenfeld residuals test generated by the cox.zph function of the R’s survival package [27] and Cox models were rejected if they did not fulfill this assumption. In this study multistate modelling was used in two ways; on one hand, a model without any proportionality assumption in baseline hazards to obtain transition-specific hazard ratios for the covariates (transition specific baseline hazards model); and, on the other hand, a model where baseline transition hazards into death are assumed to be equal (proportional baseline hazards model), to determine whether a significant effect of NAE occurrence on death exists [26].

To estimate the probability of being in each of the three different states by year from cohort entry, we first computed and depicted transition probabilities from the starting state (alive and NAE-free). Subsequently we were interested in the estimation of the differential effects of covariates on the hazard of transition among the three states. Multistate model analysis allows for simultaneous estimation of these effects considering the transition specific baseline hazards model. The crude hazard ratio (HR) for mortality of each individual NAE was taken into account for classification (see Table 1). NAEs were included in the “low-severity” category when the crude HR (95% confidence interval [CI]) for mortality was <5 (1.77–3.81); in the “intermediate-severity” category when crude HR was >5 and <15 (10.39–19.22); and in the “serious NAE” category if HR>15 (20.39–46.37). This analysis was performed for all the categories of severity of NAE, and it was also run considering three different subsets of NAE regarding NAE severity (Low, Intermediate and Serious) to obtain crude and adjusted HR and 95% CI. Adjusted HR for individual NAEs were not obtained due to the low number of transitions for some specific events.

The proportional baseline hazards model analysis was used to study specifically the effect of NAE development on the hazard of death (transition 3, “Death after NAE” versus transition 2, “Death without NAE”). In order to distinguish between transitions 2 and 3, we introduced a time-dependent covariate that indicates whether or not NAE has already occurred, named “NAE occurrence”. For transition 2 the value of this covariate equals 0, while for transition 3 the value equals 1. Adjusted HR (95% confidence interval [CI]) for all-cause mortality were calculated after having experienced a first NAE vs. remaining in the cohort NAE-free. To obtain prediction probabilities for each of the transitions in the multi-state model, we first used the previous multistate model estimated parameters and baseline transition hazards and the covariate values of a subject of interest, to obtain subject-specific transition hazards. In a second step, we used the resulting subject-specific transition hazards to obtain subject-specific transition probabilities.

**Table 1:** Incidence and outcomes of non-AIDS events classified according to severity in 8,789 people living with HIV (27,520 person-years of follow-up).

First non-AIDS event	IR per 1000pts/year (95%CI)	No. NAEs	No. deaths after NAE	Crude HR (95%CI) for death
**All**	**28.76 (26.80–30.84)**	**792**	**112**	**10.76 (8.36–13.85)**
**Low-severity NAE**	**18.74 (17.16–20.43)**	**516**	**31**	**2.60 (1.77–3.81)**
CKD stages 1 to 3*	1.52 (1.09–2.06)	42	1	1.16 (0.16–8.36)
Depression	5.81 (4.94–6.78)	160	10	2.13 (1.22–4.37)
Psychosis	0.87 (0.55–1.29)	24	1	1.39 (0.19–10.02)
Diabetes mellitus¥	2.17 (1.66–2.80)	60	1	0.53 (0.07–3.85)
Vertebral fracture	0.43 (0.22–0.76)	12	1	4.15 (0.58–29.62)
Non-vertebral fracture	2.28 (1.75–2.92)	63	2	1.37 (0.34–5.5)
Bone necrosis	0.29 (0.12–0.57)	8	0	-
Non-PJ pneumonia	5.33 (4.51–6.27)	147	15	4.56 (2.7–7.71)
**Intermediate-severity NAE**	**7.99 (6.97–9.12)**	**220**	**55**	**14.13 (10.39–19.22)**
Acute myocardial infarction & Angor	0.98 (0.64–1.42)	27	5	8.81 (3.62–21.46)
Transient ischemic attack	0.25 (0.10–0.52)	7	0	-
Acute kidney failure¶	1.48 (1.06–2.02)	41	12	10.51 (5.85–18.84)
Non-decompensated liver cirrhosis	1.74 (1.28–2.31)	48	14	12.16 (7.0–20.97)
Non-metastatic malignancy	3.52 (2.85–4.29)	97	24	13.48 (8.78–20.7)
**Serious NAE**	**2.03 (1.53–2.64)**	**56**	**26**	**30.75 (20.39–46.37)**
CKD stages 4 & 5*	0.29 (0.12–0.57)	8	2	17.8 (4.39–72.12)
Decompensated liver cirrhosis#	0.90 (0.58–1.34)	25	14	31.59 (18.34–54.43)
Stroke	0.21 (0.07–0.47)	6	2	26.48 (6.56–106.8)
Heart failure	0.29 (0.12–0.57)	8	0	**-**
Metastatic malignancy	0.32 (0.14–0.62)	9	8	**-**
**Death**	**12.78 (11.48–14.19)**	**352**	**-**	**-**

NAE, non-AIDS event; IR, incidence rate; HR, hazard ratio; CKD, Chronic Kidney Disease; PJ, *Pneumocystis jiroveci*.

*CKD stages are defined according to the KDOQI classification (stage 1, Kidney damage with normal glomerular filtration rate (GFR) [≥90 ml/min/1.73m^2^]; stage 2, Kidney damage with mild decrease in GFR [60–89 ml/min/1.73m^2^]; stage 3, Kidney damage with moderate decrease in GFR [30–59 ml/min/1.73m^2^]; stage 4, Kidney damage with severe decrease in GFR [15–29 ml/min/1.73m^2^]; stage 5, Kidney failure with very severe decrease in GFR [≤ 15 ml/min/1.73m^2^] or dialysis;

^¥^ Diabetes mellitus without end organ damage;

^¶^ 5 out of the 41 cases were patients with tubulopathy or Fanconi syndrome;

^#^ Ascites, variceal haemorrhage or hepatic encephalopathy.

#### Variables of adjustment

These covariates were used for adjustment of the transition specific baseline hazards model: sex, age at cohort entry, HIV infection category (injecting drug user, sexual, other) and the closest to cohort entry value, up to a maximum of 120 days, of AIDS diagnosis, hepatitis C coinfection, CD4-T cell count and plasma HIV-1 RNA at cohort entry covariates. The variable CD4 cell count was categorized according to the risk for the development of major opportunistic infections (> or < 200 cells/mL), and HIV-1 RNA with the cutoff that defines optimal viral suppression (< 50 copies/mL) according to the latest DHHS guidelines [28]. Hepatitis C coinfection was defined with a positive serology. To control for cohort effect, the time-period divided in two categories (2004–2008 vs 2009–2013) was also included among the covariates for adjustment. Proportional baseline hazards multistate analysis models were adjusted for severity of the event and the closest determination to the event of CD4-T cell count and HIV viral load before NAE occurrence (up to 120 days before NAE development). Interaction terms were used to evaluate potential confounding or modifying effects between variables, specifically between CD4 and RNA viral load.

## Results

Overall, 8,789 PLWH, 27,529 person-years of follow-up, were analysed. Baseline characteristics are shown in S1 Table. Table 1 represents the number of cases and distribution of the events and outcomes. Seven hundred ninety-two incident NAEs occurred during the study period in 9.01% of the study PLWH, yielding an incidence rate (95% CI) of 28.76 (26.80–30.84) per 1000 patient-years. There was a low percentage of missing data in the cohort, which was 2.4% for HIV-1 RNA, 3.2% for CD4 cell count and 3.9% for HCV status

Incidence rate of any first NAE decreased over calendar time, from 35.55 (95% CI, 31.74–39.69) cases per 1000 person-years during the period 2004–2008 to 25.53 (95% CI, 23.29–27.93) cases per 1000 person-years in the period 2009–2013 (unadjusted IRR 0.71;95%CI, 0.62–0.82). A greater decrease in the incidence rate of all-cause mortality also occurred from the first (17.34 [95% CI, 14.72–20.30] cases per 1000 person-years), to the second period (9.47, [95% CI, 8.19–10.88] cases per 1000 person-years); unadjusted IRR 0.54 (95%CI, 0.44–0.67).

**Fig 1:**
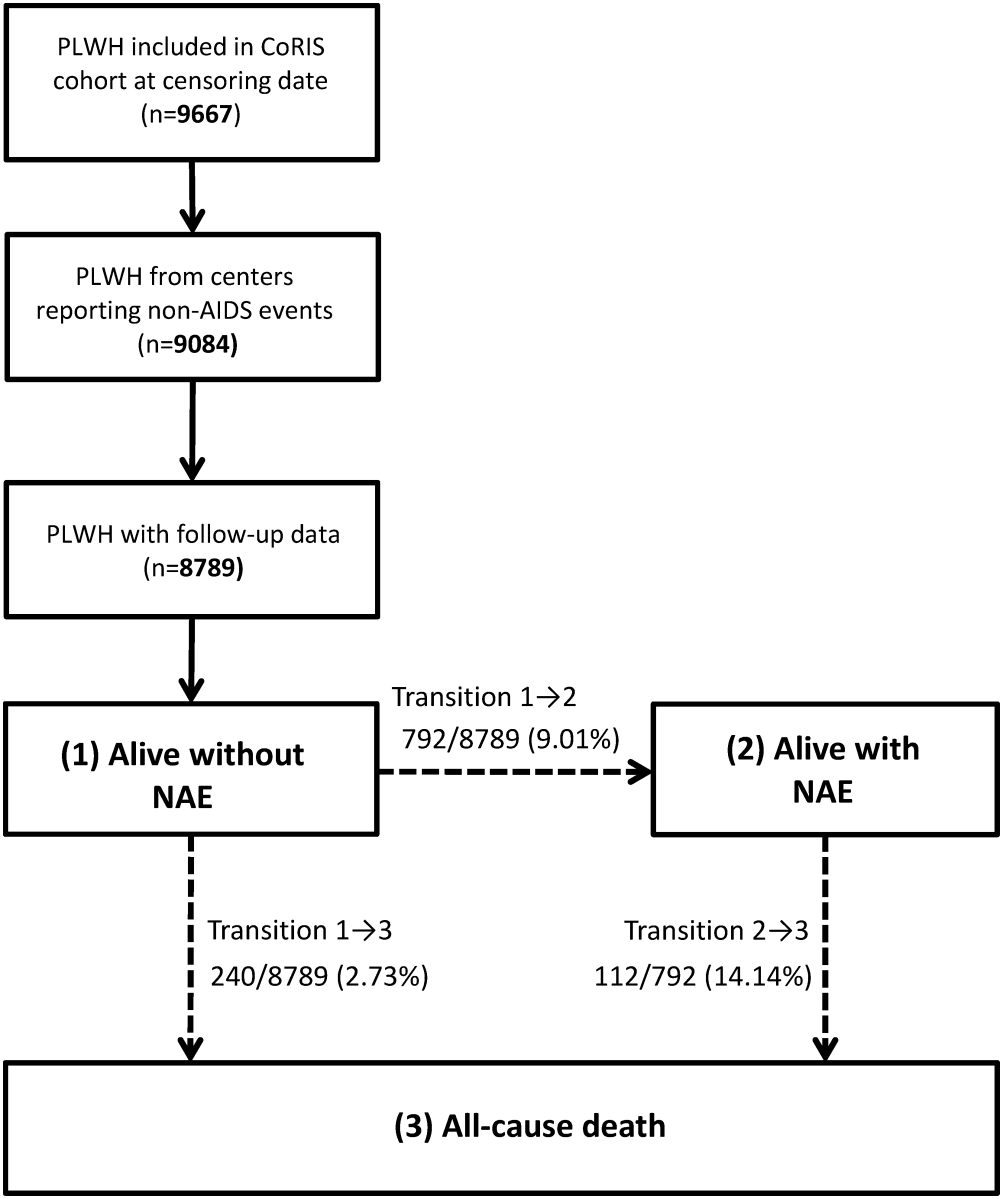
Multi-state model of non-AIDS event (NAE) development and all-cause mortality. Three possible states are considered: (1) alive without NAE, (2) alive with NAE, (3) death. Number (%) of patients for each transition; for transition 2→3 (death after NAE), the percent is calculated using the number of patients who previously reached state 2 (Alive with NAE).

**Fig 2:**
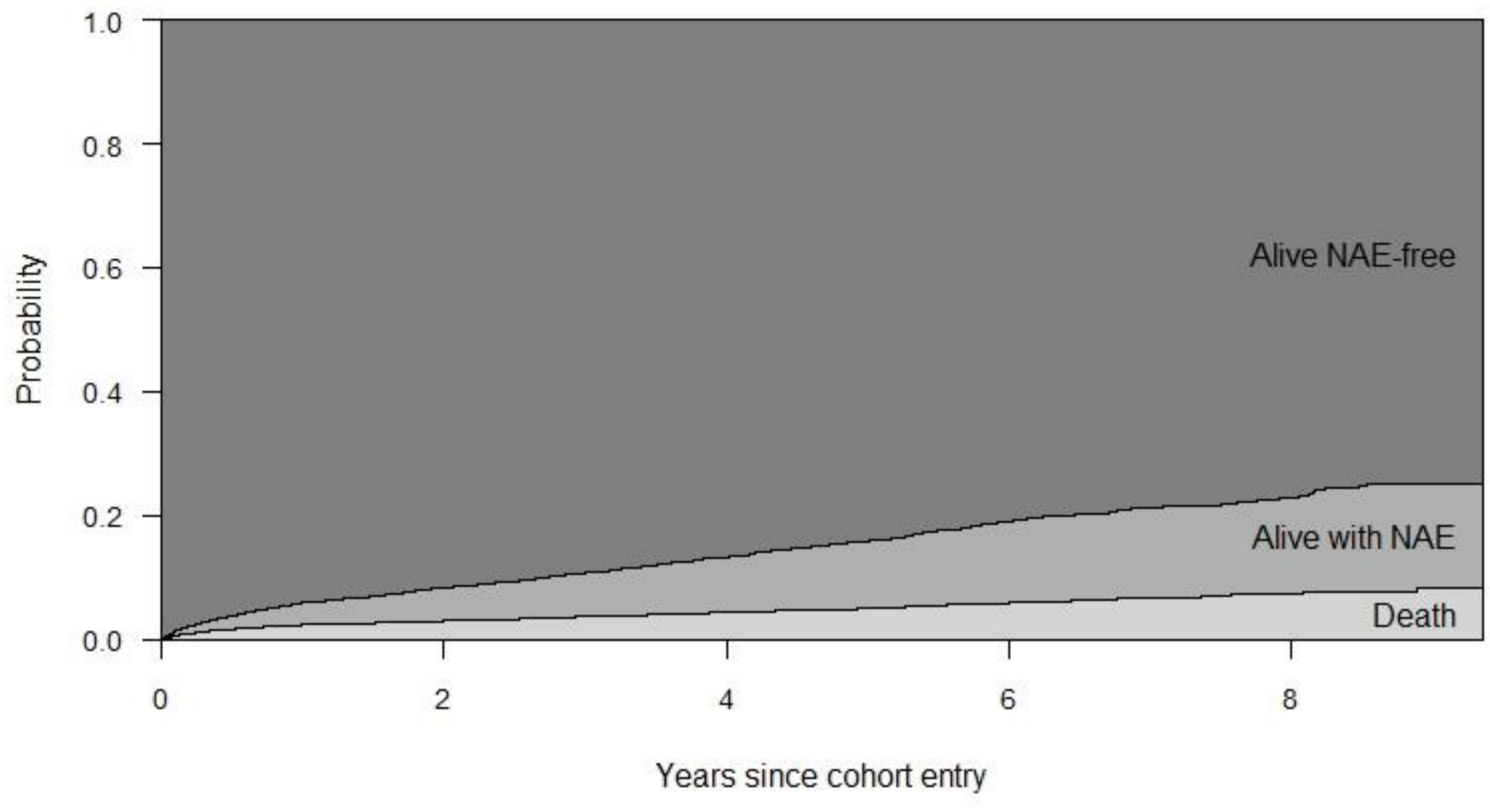
Transition probabilities between the stages along the study period. The probabilities are stacked; the distance between two curves represents the probability, associated with the text in figure. NAE, non-AIDS event.

One hundred and twelve (14.14% of NAE-experienced PLWH) finally died during follow-up. By contrast, 2.73% (240 out of 8789) of NAE-free PLWH died (Fig 1). Fig 2 represents the estimated probability of being in the states a) alive and NAE-free; b) alive and NAE-experienced; and c) dead, by year after cohort entry. For instance, 5 years after cohort entry, the estimated probability of remaining alive and NAE-free is 0.84 (standard error [SE], 0.005); of remaining alive and NAE-experienced is 0.10 (SE, 0.004); and of being dead is 0.04(SE, 0.002).

Causes of death could be identified in 301 (85.51%) PLWH; 154 (43.75%) were HIV-related deaths, followed by 44 (12.50%) non-AIDS-defining malignancies, 29 (8.23%) liver-related, 27 (7.67%) infectious and 9 (2.55%) cardiovascular diseases. A higher percentage of HIV-related death causes were found in deceased NAE-experienced PLWH (50.4% vs. 27.6%, p<0.001). Non-AIDS-defining malignancies (25.8% vs 7.5%, p<0.001) and liver-related events (14.2% vs. 8.2%, p = 0.009) were also more common in NAE-experienced PLWH.

### Classification of non-AIDS events and prognostic factors associated with mortality

Crude HR (95% CI) for mortality in NAE-experienced PLWH was 10.76 (8.36–13.85). For the low-severity category of NAE, the crude HR (95% CI) for mortality was 2.60 (1.77–3.81); for intermediate-severity, 14.13 (10.39–19.22); and for high-severity 30.75 (20.39–46.37) (Table 1).

Table 2 and S2 Table show the adjusted and crude multistate transition specific baseline hazards model with the effect of different prognostic factors on each of the three transitions, including analyses by the three different severity category subsets. Male sex (HR 2.04; 95% CI, 1.11–3.84), older age (HR 1.78; 95% CI, 1.08–2.94), lower CD4 cell count (HR 2.49; 95%CI 1.20–5.14 for CD4 cell count below 200 and HR 2.16; 95%CI 1.01–4.66 for CD4 cell count between 200–350, both compared to CD4 cell count higher than 500) and hepatitis C virus coinfection (HR 2.52; 95% CI, 1.38–4.61) showed to be prognostic factors for death after NAE development. In a sensitivity analysis including only PLWH who entered the cohort after February 2008, the same prognostic factors were found to be associated with mortality after any first NAE occurrence (data not shown). No interaction was found for CD4 cell count and HIV viral load at cohort engagement.

**Table 2:** Results of adjusted multi-state modelling prognostic factor’s effect on incident non-AIDS event (NAE) development, and on death either without or after first NAE in 8,679 people living with HIV (27,117 person-years of follow-up). Data are provided for all categories of NAEs and by severity category.

Transitions	NAE development (1→2)	Death without NAE (1→3)	Death after NAE (2→3)
	HR (95% CI)	HR (95% CI)	HR (95% CI)
**• All categories of NAE (n = 792)**			
○ Male vs female¥	0.85 (0.71–1.02)	1.25 (0.85–1.81)	2.04 (1.11–3.84)*
○ Age at cohort entry > 50 years	2.41 (1.98–2.92)**	2.49 (1.76–3.52)**	1.78 (1.08–2.94)*
○ IDU vs Sexual transmission¥	1.61 (1.23–2.12)**	1.29 (0.81–2.04)	1.38 (0.75–2.56)
○ CD4 T-cell count (cells/mL) at cohort entry			
▪ ≥500¥	1	1	1
▪ 351–499	1.03 (0.81–1.31)	0.86 (0.43–1.73)	0.86 (0.34–2.21)
▪ 200–350	1.13 (0.90–1.43)	1.62 (0.92–2.87)	2.16 (1.01–4.66)*
▪ <200	1.47 (1.18–1.84)**	3.44 (2.05–5.77)**	2.49 (1.20–5.14)*
○ Plasma HIV-1 RNA at cohort entry >10^5^ copies/ml	1.28 (1.09–1.51)*	1.46 (1.07–2.01)*	1.01 (0.66–1.56)
○ AIDS diagnosis at cohort entry	1.14 (0.92–1.41)	2.28 (1.65–3.16)**	0.82 (0.50–1.35)
○ 2004–2008 vs 2009–2013¥ period	1.40 (1.16–1.72)*	0.90 (0.64–1.25)	1.56 (0.75–3.22)
○ Hepatitis C virus coinfection	1.72 (1.34–2.21)**	3.01 (1.96–4.60)**	2.52 (1.38–4.61)**
**• Low-severity NAEs (n = 516)**			
○ Male vs female¥	0.73 (0.59–0.90)**	1.36 (0.98–1.92)	2.10 (0.80–5.55)
○ Age at cohort entry > 50 years	2.05 (1.60–2.62)**	2.46 (1.81–3.34)**	3.80 (1.78–8.11)*
○ IDU vs Sexual transmission¥	1.56 (1.10–2.14)*	1.56 (1.04–2.32)*	0.89 (0.35–2.28)
○ CD4 T-cell count (cells/mL) at cohort entry			
▪ ≥500¥	1	1	1
▪ 351–499	1.01 (0.77–1.33)	0.90 (0.49–1.66)	0.93 (0.24–3.55)
▪ 200–350	1.02 (0.78–1.34)	1.93 (1.18–3.16)*	1.82 (0.54–6.11)
▪ <200	1.19 (0.92–1.55)	3.81 (2.41–6.01)**	2.36 (0.78–7.11)
○ Plasma HIV-1 RNA at cohort entry >10^5^ copies/ml	1.34 (1.10–1.63)*	1.38 (1.05–1.80)*	1.10 (0.52–2.31)
○ AIDS diagnosis at cohort entry	1.04 (0.80–1.36)	1.86 (1.40–2.46)**	0.93 (0.40–2.15)
○ 2004–2008 vs 2009–2013¥ period	1.38 (1.09–1.75)*	1.01 (0.75–1.36)	1.88 (0.40–9.09)
○ Hepatitis C virus coinfection	1.48 (1.09–2.01)*	2.95 (2.05–4.31)**	4.59 (1.77–11.87)*
**• Intermediate-severity NAEs (n = 220)**			
○ Male vs female¥	1.07 (0.78–1.49)	1.25 (0.88–1.78)	2.79 (1.19–6.66)*
○ Age at cohort entry > 50 years	2.77 (2.02–3.79)**	2.57 (1.87–3.52)**	1.45 (0.72–2.92)
○ IDU vs Sexual transmission¥	1.45 (0.96–2.19)	1.36 (0.90–2.07)	1.68 (0.70–4.00)
○ CD4 T-cell count (cells/mL) at cohort entry			
▪ ≥500¥	1	1	1
▪ 351–499	1.08 (0.69–1.69)	0.96 (0.50–1.82)	0.43 (0.24–1.37)
▪ 200–350	1.20 (0.78–1.82)	1.90 (1.13–3.21)*	0.79 (0.30–2.03)
▪ <200	1.82 (1.23–2.70)**	3.72 (2.29–6.04)**	0.81 (0.32–2.00)
○ Plasma HIV-1 RNA at cohort entry >10^5^ copies/ml	1.23 (0.93–1.62)	1.46 (1.10–1.95)**	1.01 (0.56–1.84)
○ AIDS diagnosis at cohort entry	1.41 (1.01–1.95)*	2.00 (1.48–2.70)**	0.74 (0.40–1.38)
○ 2004–2008 vs 2009–2013¥ period	1.49 (1.04–2.08)*	0.97 (0.70–1.33)	0.78 (0.33–1.81)
○ Hepatitis C virus coinfection	2.66 (1.81–3.92)**	3.04 (2.05–4.49)**	1.68 (0.74–3.80)
**• Serious NAEs (n = 56)**			
○ Male vs female¥	1.18 (0.64–2.17)	1.41 (1.01–2.00)*	2.83 (1.03–8.33)*
○ Age at cohort entry > 50 years	3.92 (2.29–6.72)**	2.68 (1.99–3.61)**	0.59 (0.21–1.65)
○ IDU vs Sexual transmission¥	1.79 (0.88–3.62)	1.37 (0.92–2.04)	0.52 (0.14–1.94)
○ CD4 T-cell count (cells/mL) at cohort entry			
▪ ≥500¥	1	1	1
▪ 351–499	1.33 (0.52–3.37)	0.77 (0.42–1.41)	4.70 (0.44–50.2)
▪ 200–350	1.48 (0.62–3.53)	1.64 (1.02–2.64)*	5.02 (0.55–45.67)
▪ <200	2.91 (1.32–6.39)**	3.15 (2.04–4.87)**	8.65 (1.08–69.12)*
○ Plasma HIV-1 RNA at cohort entry >10^5^ copies/ml	1.34 (0.81–2.21)	1.45 (1.10–1.91)**	0.47 (0.20–1.09)
○ AIDS diagnosis at cohort entry	1.04 (0.58–1.86)	1.77 (1.33–2.36)**	1.63 (0.65–4.13)
○ 2004–2008 vs 2009–2013¥ period	3.44 (1.44–8.33)**	0.95 (0.70–1.28)	5.26 (0.49–100)
○ Hepatitis C virus coinfection	3.48 (1.75–6.93)**	3.09 (2.13–4.48)**	3.63 (1.05–12.55)*

*p≤0.05;

** p≤0.001; IDU, Intravenous drug user.

^¥^ Reference category; heterosexuals and men who have sex with men were grouped together because they share some common characteristics when confronted with intravenous drug users. For this analysis the transmission category “other/unknown” was excluded. HR, hazard ratio. The state “1” at the head of the column represents the starting status, alive and NAE-free; “2”, the transitional state, alive and NAE-experienced; and “3”, the final state of all-cause death. NAEs were classified as “low-severity” when the crude HR for mortality was <5; “intermediate-severity” when crude HR was >5 and <15; and “serious NAE” when crude HR was >15.

Prognostic factors associated with death without NAE were older age, lower CD4 cell count and higher HIV viral load both at cohort engagement, AIDS diagnosis at cohort entry and hepatitis C virus coinfection. Older age, non-sexual HIV transmission, lower CD4 cell count and higher HIV viral load at cohort engagement, hepatitis C virus coinfection and inclusion in the cohort in the earlier period (2004–2008) were independently associated with NAE development after cohort entry (Table 2).

The adjusted HR for transition to death after any first NAE occurrence in the proportional baseline hazards model analysis was 12.10 (95%CI, 4.90–29.89). There was a graded increase in the HR for mortality according to disease severity category, with an adjusted HR of 4.33 (95% CI, 2.78–6.75) for intermediate-severity NAEs, and 11.13 (95% CI, 6.60–18.79) for serious NAEs compared with low-severity NAEs. Lower CD4 cell count (<200 cells/mL) at NAE development was significantly associated with higher mortality risk (HR, 2.22; 95% CI, 1.42–3.44). No association was found between HIV viral load and mortality risk. Comparable effects were observed for concomitant CD4 cell counts and mortality for low-severity and serious NAEs. No interaction effect was found between CD4 and RNA viral load covariates in the models.

### Predicting probabilities for the outcomes

**Fig 3:**
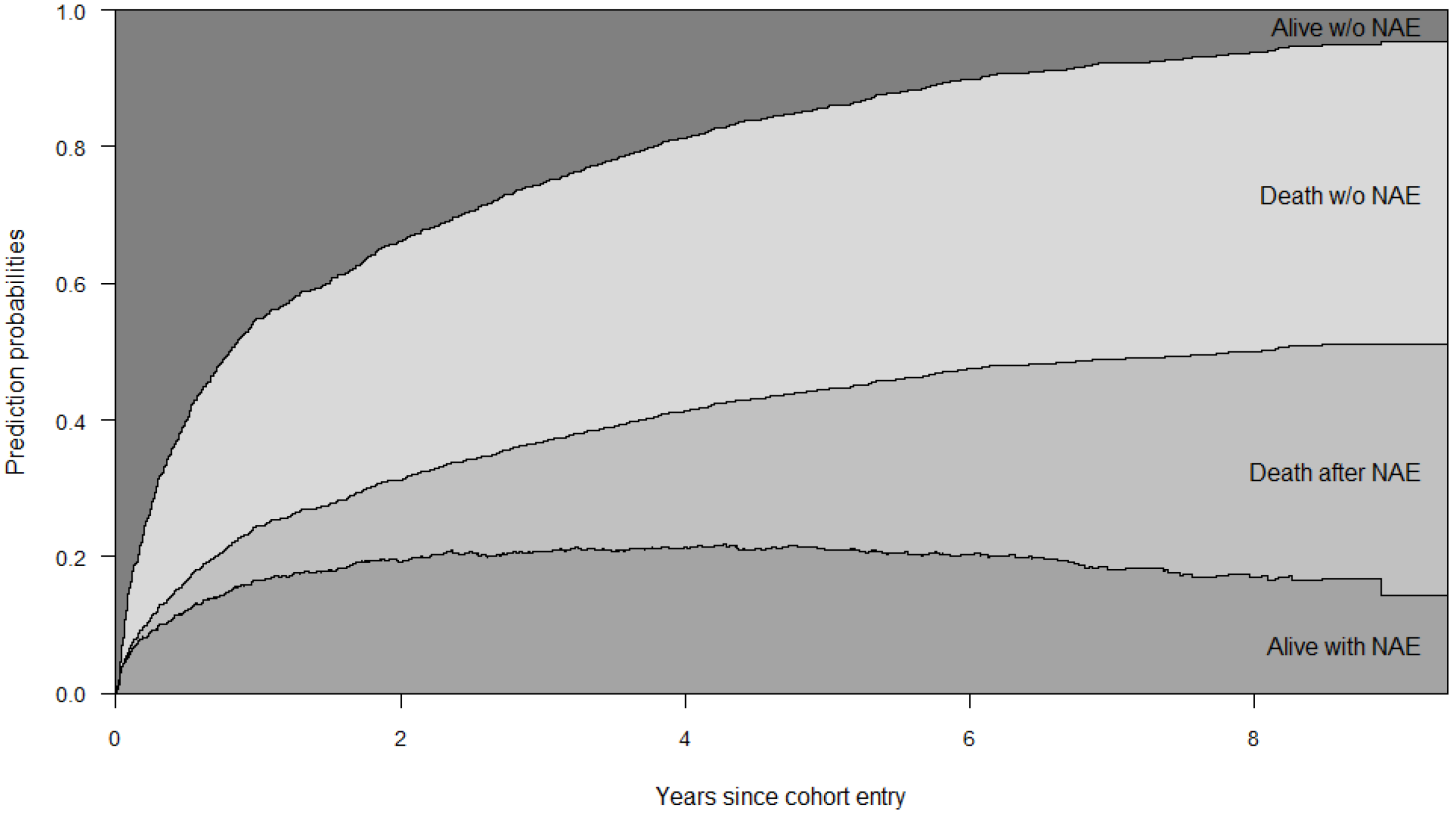
Predicted probabilities before and after non-AIDS event (NAE) development in a high risk patient (male, aged > 50 years, CD4< 200 cell/mm3 and plasma HIV1-RNA >100.000 copies/ml at engagement, IDU transmission category, previous AIDS events, cohort inclusion before 2009, hepatitis C coinfection, CD4 cell at NAE development < 500 cell/mm3 and plasma HIV1-RNA at NAE above the limit of detection). The probabilities are stacked; the distance between two curves represents the probability, associated with the text in figure.

Using the multi-state model we were able to predict probabilities for each of the 3 transitions of an individual patient according to his/her covariates values. For instance, Fig 3 shows the estimated by-year probabilities for the 3 transition rates corresponding to a “high-risk patient”: male, aged >50 years, CD4<500 cell/mL and RNA HIV >100.000 c/mL at engagement, non-sexual transmission, previous AIDS events, cohort inclusion before 2008, hepatitis C virus coinfection, CD4 cell at NAE development <500 cell/mm^3^ and RNA HIV at NAE above the limit of detection. Further tables and plots for different covariates combinations can be accessed through the following link: https://uei-elche.shinyapps.io/multistatehiv.

## Discussion

This is the first study to analyze the effect of any incident NAE occurrence on survival in PLWH. Our results show that HIV-infected people who experience a first NAE have an increased risk for all-cause mortality compared to NAE-free PLWH. There were differences between NAEs according to their impact on mortality, which allowed stratifying them into 3 categories: low-severity, intermediate-severity and serious events. Each category was associated with higher mortality risk when compared to that of NAE-free PLWH. Older age, male sex and lower concomitant CD4 cell counts were predictors of mortality after the first incident NAE development. Interestingly, different prognostic factors were found to be associated with death in NAE-free PLWH, including lower CD4 cell counts and higher HIV viral load at cohort engagement, previous AIDS diagnosis and HCV coinfection, besides older age.

With the decline in AIDS-related events and deaths after the introduction of ART, much attention has been focused on NAEs as main causes of mortality inPLWH, especially in resource-rich settings and in those with higher CD4 cell counts [29, 30]. Though classified within the same category of “events that do not meet the definition of AIDS events”, NAEs constitute a heterogeneous group of conditions involving different organ systems, with variable severity and outcomes. We suggest a classification of NAEs into three different severity categories in order to organize this mixed group of disorders, and to predict the prognosis of a patient who has experienced any first incident NAE among some of the most frequent ones. The graded increase observed in the adjusted mortality hazard from the lowest to the highest severity class constitutes the rationale for the classification and justifies its usefulness as a prognostic tool.

Outcomes following NAEs development had only been addressed in studies about severe events. However, intermediate, and mainly low-severity events do occur much more frequently than serious events inPLWH, as our results also support. Non-*Pneumocystis* pneumonia and depression, both included within the low- severity event category, were the most frequent incident NAEs in our cohort, followed by non-metastatic malignancy, which was classified as an intermediate-severity NAE. To date, no data were available regarding the prognosis of patients after any first NAE occurrence. Using multi-state model we found that NAE development increases the mortality risk in comparison to NAE-free PLWH. This finding was not only seen with serious events; low-and intermediate-severity categories were associated with a 4-fold and 22-fold, respectively, excess mortality compared with the mortality of NAE-free PLWH. We were not able to analyze individually each event because of insufficient number of transitions for some of them. However, although each severity category included events with different individual HR for mortality, all events belonging to the same category fluctuated within a similar HR range, which permitted a collective prediction for a group of events which differed from events included in alternative severity strata. Such a categorization and the information about outcomes associated with each severity stratum might contribute to expand the knowledge of the natural history of HIV infection at present, and could be useful to design the intensity of preventive and therapeutic measures to improve the long-term health of PLWH.

We found that prognostic factors for mortality differed between NAE-free and NAE-experienced PLWH. In addition to demographic data, the immunologic status when the NAE developed was a determinant factor influencing subsequent mortality. Concomitant CD4 cell count at NAE diagnosis had also been found to be a predictor of mortality in certain events, such as neoplastic and hepatic diseases [31,32]. By contrast, CD4 cell count at cohort entry was not shown to be a prognostic factor once the NAE had developed. This may support that the immunological recovery does actually modify the prognosis of PLWH,regardless of the previous immune condition. Contrary to other cohort studies, our patients were not severely immunosuppressed at cohort engagement, and this probably allowed their immune system to be satisfactorily rebuilt after initiating ART. Other factors like HIV viral load at cohort engagement, previous AIDS diagnosis, and HCV infection, were found to be predictors of the transition to death in NAE-freePLWH, but not after NAE occurrence. Those differences in the prognostic factors between PLWH either without or after NAE development support the usefulness of breaking the process of HIV infection into its different transition stages, to independently analyze the covariates effects on each state. This also enables analyzing different covariates in each transition, such as the CD4 count just before the NAE occurrence or the severity of the event for the transition from-NAE-development-to-death. Similar factors as those found in NAE-free PLWH were also predictors of any first incident NAE development after cohort engagement, mimicking the results previously described by our group and other groups [6,33,34].

Limitations of the study include the classification within the same category of different comorbid diseases with different HR for mortality, in which the category HR might not exactly match with that of each individual event. NAEs occurring before February 2008 were collected retrospectively. While underreporting cannot be excluded during the retrospective period, the incidence rate of NAEs were actually higher during the period when collection was prospective [6]. Although we included some of the most frequent NAEs, our classification did not include all possible events. In addition, this is a novel classification generated with our cohort data, which needs to be validated in other cohorts of PLWH. Although there was a different proportion of causes of death among patients who developed NAE and those who did not, this is unlikely to affect generalization of our results, as causes of death distribution in contemporary cohorts are expected to be quite similar to ours. Finally, several HRs associated with the covariates analyzed in each transition path were not statistically significant despite their effect being large, or they substantially changed after adjustment, probably because of the low number of events, along with the number of categories analyzed and collinearity between covariates. It is also worth noting that large sample sizes, as in our study, may have overestimated statistical significance of effects. Strengths are the novel classification of NAEs according to outcomes, and the use of multi-state models which allowed analyzing separately the different transition stages of the HIV disease process. Through this model we could identify the distinct outcomes and prognostic factors of each stage.

In conclusion, our study shows that NAEs occurrence increases highly the risk for mortality, and this finding was observed with each severity category, from low-severity to serious NAEs. Prognostic factors differ between NAE-free and NAE-experienced PLWH, and the immunologic status at NAE occurrence plays a central role in patients’ outcome. The suitability of this novel NAEs classification developed in our cohort will have to be validated in further cohort studies.

## Supporting information

S1 Table Baseline characteristics of the in 8,789 people living with HIV (27,520 person-years of follow-up). (DOCX)

S2 Table Results of crude multi-state modelling prognostic factor’s effect on incident non-AIDS event (NAE) development, and on death either without or after first NAE in 8,679 people living with HIV (27,117 person-years of follow-up). Data are provided for all categories of NAEs and by severity category.
(DOCX)

S1 Data Study database in csv format. (CSV)

S1 Annex Structured event reporting form containing the list of events to be reported and the precise definition of each NAE required for the inclusion. (DOC)
