# Supplementary material for: Prediction of long-term outcomes of HIV-infected patients developing non-AIDS events using a multistate approach
Source: PLoS One. 2017 Sep 8;12(9):e0184329. doi: 10.1371/journal.pone.0184329 (PMC5590896; doi:10.1371/journal.pone.0184329)
Supplement: S1 Table — (DOCX) [file pone.0184329.s001.docx]

**S1 Table. Baseline characteristics of the in 8,789 people living with HIV (27,520 person-years of follow-up).**

| **Variable** |  | | |
| --- | --- | --- | --- |
|  |  |  |  |
| **Patients**, no. |  | **8789** |  |
| **Female**, no. (%) |  | **1579** | **(17.9)** |
| **Age at cohort entry**, median years (Q_1_-Q_3_) |  | **35** | **(29-42)** |
| **HIV transmission groups**, no. (%)  Sexual  IDU  Other/Unknown |  | **7707**  **972**  **110** | **(87.7)**  **(15.2)**  **(1.2)** |
| **Prior clinical AIDS**, no. (%) |  | **983** | **(11.1)** |
| **CD4 (cells/µL) at cohort engagement**, median (Q_1_-Q_3_) |  | **368** | **(190-566)** |
| **Hepatitis C virus coinfection**, no. (%) |  | **1318** | **(14.9)** |
| **Follow-up**¥, median years (Q_1_-Q_3_) |  | **2.98** | **(1.16-5.21)** |

Q_1_-Q_3_, first and third quartiles; IDU, intravenous drug users; ¥, Years from cohort inclusion to which happened first: death, loss to follow-up or administrative censoring
