# Supplementary material for: Prediction of long-term outcomes of HIV-infected patients developing non-AIDS events using a multistate approach
Source: PLoS One. 2017 Sep 8;12(9):e0184329. doi: 10.1371/journal.pone.0184329 (PMC5590896; doi:10.1371/journal.pone.0184329)
Supplement: S2 Table — Data are provided for all categories of NAEs and by severity category. (DOCX) [file pone.0184329.s002.docx]

**S2 Table.Results of crude multi-state modelling prognostic factor’s effect on incident non-AIDS event (NAE) development, and on death either without or after first NAE in 8,679 people living with HIV (27,117 person-years of follow-up). Data are provided for all categories of NAEs and by severity category.**

| Transitions |  | NAE development  (1→2) |  | Deathwithout NAE (1→3) |  | Deathafter NAE (2→3) |
| --- | --- | --- | --- | --- | --- | --- |
|  |  | HR (95% CI) |  | HR (95% CI) |  | HR (95% CI) |
| - **All categories of NAE (n=792)** |  |  |  |  |  |  |
| - - Male vs female¥ |  | 0.81(0.68-0.96)* |  | 1.08 (0.81-1.51) |  | 2.03 (1.19-3.52)* |
| - - Age at cohort entry > 50 years |  | 2.45 (2.04-2.93)** |  | 3.20 (2.37-4.32)** |  | 1.59 (1.04-2.43)* |
| - - IDU vs Sexual transmission ¥ |  | 2.48 (2.11-2.92)** |  | 3.10 (2.33-4.11)** |  | 2.31 (1.59-3.37)** |
| - - CD4 T-cell count (cells/mL) at cohort entry |  |  |  |  |  |  |
| - - - >500¥ |  | 1 |  | 1 |  | 1 |
| - - - 351-499 |  | 1.11 (0.88-1.41) |  | 1.06 (0.55-2.06) |  | 0.98 (0.41-2.34) |
| - - - 200-350 |  | 1.40 (1.12-1.75)* |  | 2.41 (1.41-4.13)* |  | 2.06(1.02-4.16)* |
| - - - <200 |  | 2.21 (1.83-2.67)** |  | 8.65 (5.54-13.52)** |  | 2.57 (1.38-4.16)* |
| - - Plasma HIV-1 RNA at cohort entry >10^5^ copies/ml |  | 1.54 (1.34-1.78)** |  | 2.82 (2.16-3.70)** |  | 1.21 (0.82-1.77) |
| - - AIDS diagnosis at cohort entry |  | 1.81 (1.52-2.16)** |  | 6.35 (4.92-8.18)** |  | 1.31 (0.87-2.04) |
| - - 2004-2008 vs 2009-2013¥ period |  | 1.66 (1.38-2.00)** |  | 1.31 (0.99-1.75) |  | 1.92 (0.99-3.70) |
| - - Hepatitis C virus coinfection |  | 2.42 (2.08-2.88)** |  | 3.61 (2.74-4.76)** |  | 2.90 (1.92-4.26)** |
| - **Low-severity NAEs (n=516)** |  |  |  |  |  |  |
| - - Male vs female¥ |  | 0.72 (0.60-0.88)** |  | 1.21 (0.84-1.63) |  | 1.89 (0.80-4.54) |
| - - Age at cohort entry > 50 years |  | 2.01 (1.60-2.52)** |  | 3.11(2.39-4.05)** |  | 2.49 (1.28-4.48)* |
| - - IDU vs Sexual transmission¥ |  | 2.17 (1.77-2.64)** |  | 3.58 (2.82-4.55)** |  | 2.32 (1.23-4.35)* |
| - - CD4 T-cell count (cells/mL) at cohort entry |  |  |  |  |  |  |
| - - - >500¥ |  | 1 |  | 1 |  | 1 |
| - - - 351-499 |  | 1.09 (0.83-1.43) |  | 1.07 (0.60-1.89) |  | 1.15 (0.31-4.32) |
| - - - 200-350 |  | 1.22 (0.94-1.58) |  | 2.65 (1.68-4.21)** |  | 1.94 (0.60-6.03) |
| - - - <200 |  | 1.75 (1.40-2.19)** |  | 8.50 (5.45-11.88)** |  | 2.92 (1.11-7.71)* |
| - - Plasma HIV-1 RNA at cohort entry >10^5^ copies/ml |  | 1.47 (1.24-1.74)** |  | 2.51 (1.99-3.18)** |  | 1.49 (0.79-2.08) |
| - - AIDS diagnosis at cohort entry |  | 1.53 (1.22-1.91)** |  | 5.10 (4.08-6.43)** |  | 1.64 (0.82-3.28) |
| - - 2004-2008 vs 2009-2013¥ period |  | 1.53 (1.23-1.92)** |  | 1.49 (1.14-1.92)* |  | 2.04 (0.57-7.14) |
| - - Hepatitis C virus coinfection |  | 2.07 (1.72-2.49)** |  | 4.06 (3.20-5.15)** |  | 3.36 (1.79-6.32)** |
| - **Intermediate-severity NAEs (n=220)** |  |  |  |  |  |  |
| - - Male vs female¥ |  | 0.99 (0.73-1.33) |  | 1.12 (0.82-1.53) |  | 2.63 (1.25-5.55)* |
| - - Age at cohort entry > 50 years |  | 2.82 (2.11-3.78)** |  | 3.20 (2.43-4.21)** |  | 1.28 (0.74-2.19) |
| - - IDU vs Sexual transmission¥ |  | 2.85 (2.17-3.70) ** |  | 3.22 (2.5-4.16) ** |  | 1.81 (1.13-2.94) * |
| - - CD4 T-cell count (cells/mL) at cohort entry |  |  |  |  |  |  |
| - - - >500¥ |  | 1 |  | 1 |  | 1 |
| - - - 351-499 |  | 1.21 (0.78-1.86) |  | 1.12 (0.61-2.05) |  | 0.77 (0.27-2.17) |
| - - - 200-350 |  | 1.64 (1.10-2.45)* |  | 2.65 (1.62-4.34)** |  | 1.16 (0.50-2.70) |
| - - - <200 |  | 3.06 (2.19-4.28)** |  | 8.47 (5.57-12.88)** |  | 1.15 (0.56-2.37) |
| - - Plasma HIV-1 RNA at cohort entry >10^5^ copies/ml |  | 1.65 (1.29-2.11)** |  | 2.70 (2.10-3.45)** |  | 0.92 (0.57-1.50) |
| - - AIDS diagnosis at cohort entry |  | 2.38 (1.79-3.14)** |  | 5.54 (4.37-7.03)** |  | 0.86 (0.50-1.48) |
| - - 2004-2008 vs 2009-2013¥ period |  | 1.92 (1.38-2.70) ** |  | 1.40 (1.07-1.85) * |  | 0.97 (0.45-2.08) |
| - - Hepatitis C virus coinfection |  | 3.40 (2.65-4.37)** |  | 3.38 (2.98-4.92)** |  | 2.06 (1.26-3.37)* |
| - **Serious NAEs (n=56)** |  |  |  |  |  |  |
| - - Male vs female¥ |  | 1.14 (0.67-2.0) |  | 1.24 (0.92-1.69) |  | 1.53 (0.68-3.57) |
| - - Age at cohort entry > 50 years |  | 3.78 (2.36-6.05)** |  | 3.22 (2.48-4.18)** |  | 0.91 (0.43-1.89) |
| - - IDU vs Sexual transmission¥ |  | 4.54 (3.03-7.14)** |  | 3.12 (2.43-4.00)** |  | 1.63 (0.89-3.03) |
| - - CD4 T-cell count (cells/mL) at cohort entry |  |  |  |  |  |  |
| - - - >500¥ |  | 1 |  | 1 |  | 1 |
| - - - 351-499 |  | 1.53 (0.64-3.69) |  | 0.96 (0.55-1.69) |  | 3.13 (0.56-17.25) |
| - - - 200-350 |  | 1.95 (0.85-4.47) |  | 2.35 (1.50-3.67)** |  | 3.17 (0.66-15.09) |
| - - - <200 |  | 5.24 (2.65-10.37)** |  | 6.99 (4.81-10.16)** |  | 4.26 (1.08-19.67)* |
| - - Plasma HIV-1 RNA at cohort entry >10^5^ copies/ml |  | 1.89 (1.23-2.89)* |  | 2.59 (2.05-3.28)** |  | 0.83 (0.45-1.55) |
| - - AIDS diagnosis at cohort entry |  | 2.27 (1.39-3.71)** |  | 4.98 (3.97-6.26)** |  | 1.02 (0.54-2.01) |
| - - 2004-2008 vs 2009-2013¥ period |  | 4.54 (2.12-10.0)** |  | 1.35 (1.04-1.78)* |  | 5.00 (0.65-50.0) |
| - - Hepatitis C virus coinfection |  | 5.49 (3.57-8.43)** |  | 3.70 (2.94-4.74)** |  | 2.95 (1.50-5.79)* |

*p≤0.05; ** p≤0.001; IDU, Intravenous drug users. ¥ Reference category; heterosexuals and men who have sex with men were grouped together because they share some common characteristics when confronted with intravenous drug users. For this analysis the transmission category “other/unknown” was excluded. HR, hazard ratio. The state “1” at the head of the column represents the starting status, alive and NAE-free; “2” , the transitional state, alive and NAE-experienced; and “3”, the final state of all-cause death. NAEs were classified as “low-severity” when the crude HR for mortality was <5; “intermediate-severity” when crude HR was >5 and <15; and “serious NAE” when crude HR was >15.
